# Comparison of voluntary food intake and palatability of commercial weight loss diets in healthy dogs and cats

**DOI:** 10.1186/s12917-016-0899-x

**Published:** 2016-12-05

**Authors:** Marie Anne Hours, Emmanuelle Sagols, Ariane Junien-Castagna, Alexandre Feugier, Delphine Moniot, Ingrid Daniel, Vincent Biourge, Serisier Samuel, Yann Queau, Alexander J. German

**Affiliations:** 1 Royal Canin Research Center, Aimargues, France; 2 Unité de Nutrition et d’Endocrinologie, Oniris, Nantes, France; 3 Institute of Ageing and Chronic Disease, University of Liverpool, Leahurst Campus, Chester High Road, Neston, Wirral CH64 7TE UK; 4 Université de Nantes, Faculty of Dental Surgery, 1 Place Alexis Ricordeau, 44042 Nantes, France; 5 Institute of Veterinary Science, University of Liverpool, Leahurst Campus, Chester High Road, Neston, CH64 7TE UK

**Keywords:** Obesity, Canine, Appetite, Weight loss

## Abstract

**Background:**

Obesity in dogs and cats is usually managed by dietary energy restriction using a purpose-formulated weight loss diet, but signs of hunger and begging commonly occur causing poor owner compliance. Altering diet characteristics so as to reduce voluntary food intake (VFI) can improve the likelihood of success, although this should not be at the expense of palatability. The aim of the current study was to compare the VFI and palatibility of novel commercially available canine and feline weight loss diets.

**Methods:**

The relative performance of two canine (C1 and C2) and two feline (F1 and F2) diets was assessed in groups of healthy adult dogs and cats, respectively. Diets varied in energy, protein, fibre, and fat content. To assess canine VFI, 12 (study 1) and 10 (study 2) dogs were offered food in 4 meals, for 15 min on each occasion, with hourly intervals between the meals. For feline VFI, 12 cats were offered food ad libitum for a period of 18 h per day over 5 consecutive days. The palatability studies used separate panels of 37 dogs and 30 cats, with the two diets being served, side-by-side, in identical bowls.

**Results:**

In dogs, VFI was significantly less for diet C1 than diet C2 when assessed on energy intake (study 1, 42% less, *P* = 0.032; study 2, 28% less, *P* = 0.019), but there was no difference in gram weight intake (study 1: *P* = 0.964; study 2: *P* = 0.255). In cats, VFI was 17% less for diet F1 than diet F2 when assessed by energy intake (*P* < 0.001), but there was again no difference in gram weight (*P* = 0.207). There was no difference in palatability between the two canine diets (*P* = 0.490), whilst the panel of cats diet preferred F1 to F2 (*P* < 0.001).

**Conclusion:**

Foods with different characteristics can decrease VFI without affecting palatability in both dogs and cats. The effects seen could be due to decreased energy content, decreased fat content, increased fibre content, different fibre source, and increased protein content. Further studies are now needed to determine whether similar findings occur in obese dogs and cats on controlled weight loss programmes.

**Electronic supplementary material:**

The online version of this article (doi:10.1186/s12917-016-0899-x) contains supplementary material, which is available to authorized users.

## Background

Obesity is now a common medical disorder in both dogs and cats, and has various effects on the health of animals of both species [1–5]. Controlled weight loss has been shown to have a number of benefits in previously obese dogs, including improved mobility [6], improved respiratory function [4], resolution of metabolic disturbances [7, 8], and improved quality of life [5]. Dietary energy restriction using a purpose-formulated diet is the most common approach for inducing weight loss, and such strategies are usually very successful in experimental trials in both dogs [9–11] and cats [12, 13]. However, the same strategies do not perform as well in a clinical setting, for obese client-owned pets, with slower rates of weight loss observed despite marked energy restriction [14–17]. Further, many dogs and cats do not successfully reach their target weight, and this is most often because owners struggle to comply with the programme ultimately deciding to stop [18, 19]. A common problem that owners encounter is the fact that dietary energy restriction causes hunger, which causes increased begging and scavenging activity in their dog or cat. Such behaviour can be difficult for the owner to resist, ultimately leading to poor compliance. Indeed, recent studies have indicated that many owners feed additional food during a controlled weight loss programme despite veterinary recommendations [14, 15].

Food manufacturers can alter a range of dietary characteristics, and such changes can affect voluntary food intake (VFI). For example, a weight management diet can be changed so as to reduce VFI, and such a modification should increase the likelihood of success, provided that it does not adversely affect palatability and, therefore, overall diet acceptance. Approaches that can be used in dogs and cats include decreasing nutrient density, for instance by expanding kibble volume with air [20] or water [21], and altering the macronutrient content of the diet by increasing protein and/or fibre content [22, 23]. In addition to caloric dilution, adding dietary water can increase voluntary physical activity and may have added benefits for weight loss [21]. With regard to macronutrient content, recent studies have indicated that a diet containing increased amounts of both protein and fibre are more effective at reducing VFI than diets containing increased amounts of these macronutrients individually [22], and have shown that such diets lead to improved outcomes of weight loss in obese pet dogs [17]. In cats, the ideal balance of protein and fibre is more difficult to optimise because very high protein diets can actually stimulate VFI in cats, whilst very high fibre diets can be unpalatable [23]. Despite this, dry diets that combine moderately increased protein and fibre content are better at reducing begging activity in obese cats during a controlled weight loss programme [16].

Given the importance of obesity as a medical disease, and the recognition that current strategies are not perfect [18], there has been a great deal of recent interest in improving diets for controlled weight loss so as to improve outcomes. Indeed, in the last five years, new diets have been developed and become commercially available [24, 25], and many existing commercial weight loss diets have been reformulated [18]. As a result, there is a need to assess the efficacy of diets that are currently available. Therefore, the aim of the current study was to compare the performance, in terms of VFI and palatability, of novel commercially-available canine and feline weight loss diets, in groups of healthy dogs and cats housed in research colonies.

## Methods

### Research sites and study animals

The studies were undertaken between January 2014 and July 2014 at two sites: the Royal Canin Research Center, Aimargues, France (Site 1), and the National Veterinary School of Nantes, Food Science and Engineering, (ONIRIS) France (Site 2). The first canine VFI study, the feline VFI study, and both the feline and canine palatability studies were all performed at site 1; the second canine VFI study was performed at site 2. The participating cats and dogs were colony animals; those from site 1 were sourced from private breeders, whilst those from site 2 were born and raised at research site itself. All animals were deemed to be healthy prior to the start of the study, based upon health checks (comprising physical examination), and clinicopathological assessments (e.g. blood chemistries and complete blood counts), conducted on a monthly and annual basis, respectively. All remained healthy during the studies, with no adverse events were reported, and no modifications to any of the experimental protocols were required. Faecal consistency also remained throughout, albeit a greater volume was consistently produced on the test diets given the increased fibre content.

The first canine VFI study was undertaken in May 2014 and involved twelve healthy neutered female adult small breed dogs (5 Miniature Schnauzers, 5 Bichon Frisés, 1 Miniature Dachshund and 1 Cairn terrier), in ideal body condition (body condition score [BCS] 5/9), with a median age of 6y 8mo (range 3y 10mo to 13y 0mo). The second canine VFI study was undertaken in June 2014 and involved ten healthy beagle dogs (4 neutered females, 6 intact males) in ideal body condition (BCS 5/9), with a median age of 4y 3mo (range 2y 8mo to 6y 0mo). The feline VFI study was undertaken in May 2014 and involved 12 healthy adult cats (7 neutered males and 5 neutered females), with a median age of 4y 1mo (range 4y 0mo to 4y 3mo). Nine of the cats were of the domestic shorthair breed, whilst the remaining 3 were Bengal. Median body condition score was 4/9 (range 4–8/9), with 10 cats being in ideal weight (BCS 4-5/9) and 2 cats being overweight (BCS 6/9 and 8/9).

The dog palatability study was undertaken in January 2014 and involved 37 healthy neutered female adult dogs (median age, 2y 10mo, range 1y 2mo to 11y 5mo) from various breeds including: Beauceron (1), Bernese Mountain Dog (2), Brittany Spaniel (1), Cairn Terrier (2), Cocker Spaniel (4), Dachshund (4), English Setter (2), Flat Coated Retriever (1), German Shepherd Dog (4), German Wirehaired Pointer (2), Gordon Setter (2), Irish Setter (1), Jack Russell Terrier (7), Miniature Schnauzer (1), Portuguese Podengo (1), and West Highland White Terrier (2). The cat palatability study was undertaken in July 2014 and involved 30 healthy adult cats (17 neutered females, 13 neutered males), with a median age of 7y 0mo (range 3y 4 mo to 14y 5 mo), from various breeds including: Abyssinian (1), Bengal (2), Birman (4), Chartreux (1), Domestic Shorthair (12), Exotic Shorthair (2), Maine Coon (2), Oriental (1), Siamese (1), Somali (3), and Sphynx (1).

### Housing and husbandry

Housing and treatment protocols adhered to European regulatory rules for animal welfare. At site 1, dogs were housed in groups of two in closed indoor-outdoor runs, the size of which varied depending upon the size of the dogs (indoor box size: 5.4-9.3 m^2^; outdoor run size: 3.6-12.5 m^2^). For the feeding studies, all dogs were fed individually, using separate ‘traps’ within their own pen. At site 2, dogs were housed in groups of 6 in outdoor runs of 20 m^2^, with half of the run being covered. Dogs also had free access to dog houses of 1.9 m^2^ (Dogloo® X-Large, Petmate, Arlington, USA). For the feeding studies, dogs were again fed individually, this time using individual pens of 4 m^2^. Cats were group-housed in closed indoor-outdoor runs, of 27 m^2^, with a maximum of 8 cats per run. The runs with outdoor access were divided into an indoor part (of 13 m^2^) and an outdoor part (of 14 m^2^). For the feeding studies, cats were fed using automated feeding stations (see below). Dependent on the season, the inside temperature varied between 18 °C and 24 °C. For both dog and cat housing at site 1, artificial light was provided in addition to the natural light, between 07.30 and 17.00, if natural light was judged to be insufficient by the animal caregivers. This was not the case for site two because of the use of outdoor runs. All dogs had exercise sessions of 2 h/day at site 1 and at least 1 h/day at site 2. For cats, caregivers stimulated play behaviour for approximately 2 h per run, per day.

### Diets

The VFI and palatability studies involved four complete and balanced diets, purpose-formulated for weight loss, two designed for feeding to dogs, and two for cats (Table 1). Diet C1 was a high protein high fibre diet (Satiety Weight Management Canine, Royal Canin, Aimargues, France), whilst diet C2 was a moderate protein high fibre diet (Prescription Diet® Canine Metabolic Advanced Weight Solution, Hill’s Pet Nutrition, Topeka, KS, USA). These two diets differed in energy content (average dietary composition based upon typical analysis: C1, 12041 KJ/kg [2876 kcal/kg]; C2, 12996 KJ/kg [3104 kcal/kg]) and macronutrient profile, with diet C1 containing more protein (104 g/1000 kcal vs. 84 g/1000 kcal) and fibre (crude fibre: 58 g/1000 kcal vs. 43 g/1000 kcal), but less fat (33 g/1000 kcal vs. 37 g/1000 kcal) and nitrogen-free extract (NFE 101 g/1000 kcal vs. 113 g/1000 kcal) than diet C2 (Table 1).

**Table 1:** Average dietary composition based upon typical analysis of the 4 diets assessed used during the study

Criterion	Diet C1		Diet C2		Diet F1		Diet F2	
Species	Dog		Dog		Cat		Cat	
ME content^a^	12041 KJ/kg 2876 kcal/kg		12996 KJ/kg 3104 kcal/kg		12405 KJ/kg 2963 kcal/kg		14302 KJ/kg 3416 kcal/kg	
	Per 100 g AF	g/1000 kcal	Per 100 g AF	g/1000 kcal	Per 100 g AF	g/1000 kcal	Per 100 g AF	g/1000 kcal
Moisture	9.5	33	8.5	27	5.5	19	5.5	18
Protein	30	104	26	84	34	118	37.7	121
Fat	9.5	33	11.4	37	9	31	12.8	41
Crude fibre	16.6	58	13.4	43	13.9	48	9.1	29
TDF	28.1	98	23.8	77	23.6	82	16.6	53
NFE	29.1	101	35	113	28.8	100	28.8	93
Ash	5.3	18	5.7	18	8.8	31	6.1	20
Ingredients	Vegetable Fibres, Dehydrated Poultry Protein, Wheat Gluten, Tapioca, Maize Gluten, Hydrolysed Animal Proteins, Maize, Wheat, Animal Fats, Beet Pulp, Fish Oil, Minerals, Fructo-Oligo-Saccharides, Soya Oil, Psyllium Husks and Seeds, Hydrolysed Crustaceans, Marigold Extract, Hydrolysed Cartilage; Vitamin A, Vitamin D3, E1 (Iron), E2 (Iodine), E4 (Copper), E5 (Manganese): E6 (Zinc), E8 (Selenium), Preservatives, Antioxidants		Chicken By-Product Meal, Whole Grain Wheat, Whole Grain Corn, Corn Gluten Meal, Pea Bran Meal, Soybean Meal, Soybean Mill Run, Dried Tomato Pomace, Chicken Liver Flavour, Dried Beet Pulp, Flaxseed, Coconut Oil, Pork Fat, Lactic Acid, Powdered Cellulose, Pork Liver Flavor, DL-Methionine, L-Lysine, Iodized Salt, Dried Carrots, Dicalcium Phosphate, Potassium Chloride, Vitamin E Supplement, L-Ascorbyl-2-Polyphosphate, Niacin Supplement, Thiamine Mononitrate, Vitamin A Supplement, Calcium Pantothenate, Biotin, Vitamin B12 Supplement, Pyridoxine Hydrochloride, Riboflavin Supplement, Folic Acid, Vitamin D3 Supplement, Lipoic Acid, Choline Chloride, Manganese Sulphate, Ferrous Sulphate, Zinc Oxide, Copper Sulphate, Calcium Iodate, Sodium Selenite, Taurine, Mixed Tocopherols, L-Carnitine, Beta-Carotene, Phosphoric Acid, Natural Flavours		Dehydrated Poultry Meat, Vegetable Fibres, Tapioca, Wheat Gluten, Wheat Flour, Maize Gluten, Hydrolysed Animal Proteins, Animal Fats, Minerals, Chicory Pulp, Fish Oil, Psyllium Husks and Seeds, Hydrolysed Crustaceans, Marigold Extract, Hydrolysed Cartilage, Vitamin A, Vitamin D3, E1 (Iron), E2 (Iodine), E4 (Copper), E5 (Manganese), E6 (Zinc), E8 (Selenium), Preservatives, Antioxidants		Chicken By-Product Meal, Brewers Rice, Corn Gluten Meal, Powdered Cellulose, Dried Tomato Pomace, Flaxseed, Dried Beet Pulp, Chicken Liver Flavor, Coconut Oil, Pork Fat, Lactic Acid, Potassium Chloride, Calcium Sulfate, L-Lysine, Choline Chloride, Carrots, DL-Methionine, Taurine, vitamins (Vitamin E Supplement, L-Ascorbyl-2-Polyphosphate (source of vitamin C), Niacin Supplement, Thiamine Mononitrate, Vitamin A Supplement, Calcium Pantothenate, Pyridoxine Hydrochloride, Riboflavin Supplement, Biotin, Vitamin B12 Supplement, Folic Acid, Vitamin D3 Supplement), minerals (Manganese Sulfate, Ferrous Sulfate, Zinc Oxide, Copper Sulfate, Calcium Iodate, Sodium Selenite), L-Carnitine, Mixed Tocopherols, Beta-Carotene, Phosphoric Acid, Natural Flavours	

^a^ Metabolisable energy content for each diet was calculated using Modified Atwater factors, based on the declared average dietary composition information for each diet. The effect of possible batch variation was not taken into account. *AF* as fed, *NFE* nitrogen free extract, *TDF* total dietary fibre. Diet C1: Satiety Weight Management Canine, Royal Canin, Aimargues, France; Diet C2: Prescription Diet® Canine Metabolic Advanced Weight Solution, Hill’s Pet Nutrition, Topeka, KS, USA; diet F1: Satiety Weight Management Feline, Royal Canin, Aimargues, France; Diet F2: Prescription Diet® Metabolic Feline, Hill’s Pet Nutrition, Topeka, KS, USA

The ingredients used also varied, including fibre sources (C1: vegetable fibres, beet pulp and psyllium [husks and seeds]; C2: pea bran meal, tomato pomace, beet pulp, and powdered cellulose). The remaining two diets were designed for feeding to cats (diet F1: Satiety Weight Management Feline, Royal Canin Aimargues, France; Diet F2: Prescription Diet® Metabolic Feline, Hill’s Pet Nutrition Topeka, KS, USA). Protein content was similar between diets (diet F1: 118 g/1000 kcal, diet F2: 121 g/1000 kcal), but diet F1 contained more fibre (crude fibre: F1, 48 g/1000 kcal; F2, 29 g/1000 kcal; total dietary fibre: F1, 82 g/1000 kcal; C2, 53 g/1000 kcal) and NFE (F1: 100 g/1000 kcal; F2: 93 g/1000 kcal), and less fat (31 g/1000 kcal vs. 41 g/1000 kcal), than diet F2. Dietary energy content was also less in diet F1 (F1: 12405 KJ/kg [2963 kcal/kg]) than in diet F2: (14302 KJ/kg [3416 kcal/kg]). Again, ingredients varied amongst diets, most notably for fibre source (F1: vegetable fibres, chicory pulp, and psyllium [husks and seeds]; F2: powdered cellulose, tomato pomace, and beet pulp).

Finally, organoleptic properties of the diets also varied amongst diets, with differences including shape,colour, texture, and smell. Diets C1 and F1 had a round (pastille) shape, whilst diets C2 and F2 had a triangular prism shape. All diets were brown in colour, with the shade being marginally lighter for diets C2 and F2 compared with diets C1 and F1, respectively. None of diets were enriched with artificial colourings.

### Canine VFI studies

**Fig. 1:**
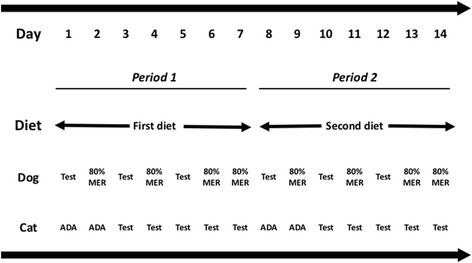
Summary of the trial design for the voluntary food intake studies. For both canine studies, dogs were fed each diet, sequentially, for periods of 7 days. The test protocol (Test) was performed on 3 non-consecutive days for each study period, with food intake being limited to 80% of MER (e.g. study 1: 88 Kcal/kg^0.75^; study 2: 96 Kcal/kg^0.75^). For the feline voluntary food intake study, cats were fed each diet *ad libitium*, sequentially, for periods of 7 days, with each an initial 2-day adaptation phase (ADA) and then a 5-day test phase (Test)

Two studies were performed to determine VFI, with the first study using dogs from site 1 and the second study using dogs from site 2. The design of each study was the same, except that different methods were used for calculating the metabolisable energy required for maintenance (MER; study 1: 110 Kcal/kg^0.75^/day; study 2: 120 Kcal/kg^0.75^/day), given differences in the known MER of each group. In each study, dogs were fed the two diets (C1 and C2) for a period of 7 days, using a crossover design (Fig. 1), with half of the dogs receiving diet C1 first, and the other half receiving diet C2 first. The order of the diets was arbitraily decided in advance by the researchers, but did not used a formal method of randomisation. In order to minimise unwanted weight gain, the test protocol was performed on 3 non-consecutive days for each study period whilst, on the non-study days, food intake was reduced to 80% of MER (e.g. study 1: 88 Kcal/kg^0.75^; study 2: 96 Kcal/kg^0.75^). The two periods ran consecutively, with no adaptation period between diets. However, prior to the start of each study, all dogs had been offered both foods to familiarise them. On test days, consumption kinetics was assessed through repeated short-term food exposure, using a modification of a protocol previously described [20, 22]. Briefly, each dog was offered 110 kcal/kg^0.75^ for 15 min at 08:30 (1^st^ meal) and again at 09:30 (2^nd^ meal), and then offered food ad libitum for 15 min at both 10:30 (3^rd^ meal) and 11:30 (4^th^ meal). At all meals, dogs left the bowl before the end of the 15-min feeding period, with most finishing eating within 5 min. Water was freely available for consumption at all times. Food intake was measured by weighing the bowl on calibrated electronic gram scales (Site 1: P8000-S, Mettler-Toledo, Albstadt, Germany; Site 2: NVT 160 000, OHAUS, Nänikon, Switzerland; both scales accurate to within 1 g) before and after each meal to determine the amount of food eaten.

Body weight (BW) was recorded on a weekly basis throughout the trial period using calibrated electronic weigh scales (Site 1: SG16000, Mettler Toledo; Site 2: SPIDER SW, Mettler Toledo, accurate to within 50 g), and the mean bodyweight for this period was used to calculate the mean study metabolic body weight (MBW, e.g. BW^0.75^ in kg; NRC 2006). Energy intake at each meal was then calculated by multiplying the energy content of the food by the amount consumed, and then dividing this by the dog’s average study MBW.

### Feline VFI study

**Fig. 2:**
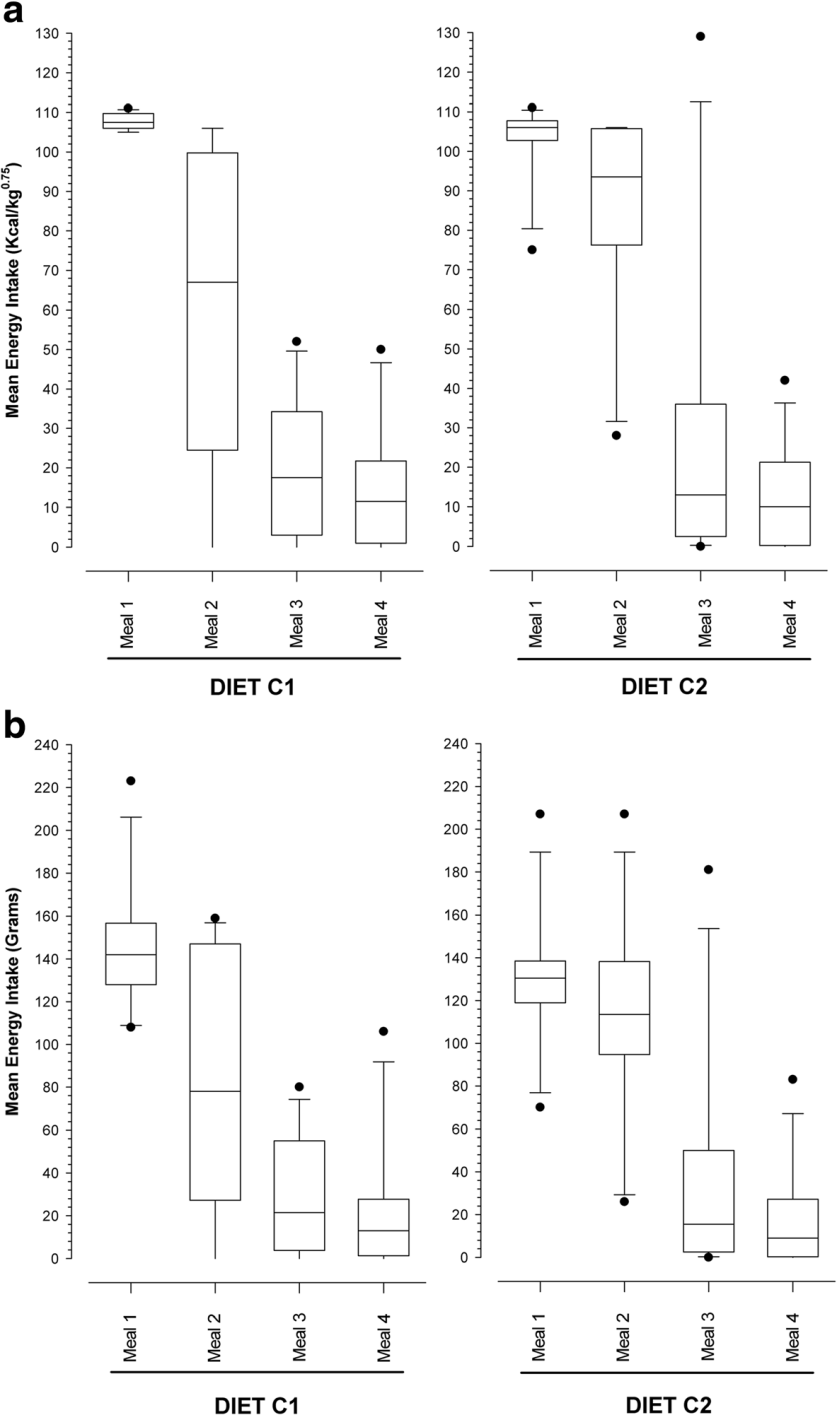
Box and whisker plots of sequential energy (**a**) and gram weight (**b**) intake in the first canine voluntary food intake study (Study 1) where dogs were fed the two study diets (C1 and C2), over four meals. The boxes depict median (horizontal line) and inter-quartile range (top and bottom of box), the whiskers show the 10–90% range, and outliers are shown as separate points. Each dog was offered 110 kcal/kg^0.75^ for 15 min at 08:30 (1^st^ meal) and again at 09:30 (2^nd^ meal), and then offered food ad libitum for 15 min at both 10:30 (3^rd^ meal) and 11:30 (4^th^ meal). **a** A significant reduction of energy intake was observed between the second and third meals for both diets (*P* < 0.001), but between the first and second meals for diet C1 only (C1: *P* < 0.001; C2: *P* = 0.256). A diet effect was also evident (*P* = 0.032), with the main difference being a lesser intake at meal two for C1 compared with C2 (*P* = 0.006). **b** A significant reduction in gram weight intake of food was observed between the second and third meals for both diets (*P* < 0.001), but between the first and second meals for diet C1 only (C1: *P* < 0.001; C2: *P* = 0.960). However, no difference in the gram weight intake of food was observed between diets (*P* = 0.964)

As with the canine study, cats were fed the two diets (F1 and F2), each for periods of 7 days, again using a crossover design (Fig. 2), with half of the cats receiving diet F1 first, and the other half receiving diet F2 first. Again, the order of the diets was arbitrarily decided in advance by the researchers. Each period consisted of an initial 2-day adaptation phase, and then a 5-day test phase. On each test day, the respective diet was offered ad libitum for a period of 18 h, with no food being available for the remaining 6-h so as to limit excessive food consumption during the study. The period of food availability (between 14:00 and 08:00 on each test day) was selected to ensure that food was available for the known times of peak consumption within the colony (i.e. during the evening and early hours of the morning), and also fitted best with the daily routines of the animal caregivers. Water was freely available for consumption throughout the study. Each cat had access to its own food station by microchip recognition, and individual food intake (in grams) was recorded daily using electronic weigh scales (M-Tronic Paris; France; accurate to within 0.5 g). Energy intake was then calculated by multiplying the energy content of the food by the amount consumed.

As with the canine study, body weight was recorded on a weekly basis throughout the study period using calibrated weigh scales SG16000; Mettler Toledo), and the mean body weight for the whole period used to calculate the mean study MBW (e.g. BW^0.711^ in kg; NRC 2006). Each cat’s food energy intake was then expressed relative to MBW.

### Canine and feline palatability studies

For the canine palatability study, a panel of 37 entire female dogs participated, all of which were routinely used in palatability testing at site 1. A range of different sizes, breeds and ages were represented. The protocol was repeated on 2 consecutive meals on the same day, at 08:00 and 16:00 (M1, M2). For each test, the two diets were served, side-by-side in identical bowls, with the food allocated to each bowl arbitrarily decided. The amount provided in each bowl was equivalent to twice the energy requirements recommended for each dog. At the end of the 15-min test period, the amount of each food consumed by all dogs was measured.

A similar approach was chosen for the feline palatability study, although a panel of 30 cats participated. Again, this panel was routinely used for palatability testing, and a range of breeds, ages and genders was represented. The protocol was performed twice on two consecutive days, such that both diet (F1 vs. F2) and day (D1 vs. D2) effects were assessed. As with the canine study, the two diets were served, side-by-side in two identical bowls, with the food allocated to each bowl again arbitrarily determined. The amount of each food provided was equivalent to twice the energy requirements recommended for each cat. However, cats had free access to both diets over a 22-h-period (i.e. from 10:00 until 08:00). Food intake of both diets was again recorded using the same approach as for the canine palatability study.

### Data handling and statistical analysis

The sample sizes decided for the studies were not determined by use of a power analysis calculation. Instead, the group size used was equivalent to that used in previous studies assessing VFI and palatability [20, 22]. For the VFI studies, the primary outcome measure of interest was the amount of energy consumed (expressed both as KJ and Kcal per kg of MBW), whilst secondary outcomes included the weight of food consumed (in grams), and also BW (in kg) measured before and after each protocol (as described above). For the palatability studies, the primary outcome measure was the amount of each diet consumed in grams.

In all studies, complete data were available for all animals participating, except for one cat in the Feline VFI study whereby malfunction of the electronic food scales meant that the data could not be used. Data were recorded in a computer spreadsheet (Additional file 1; Excel For Mac version 15.28, Microsoft Inc.) and analysed using the Statistical Analysis Systems institute package (SAS version 9; SAS Institute Inc.). For the canine VFI, a linear mixed model assessing the fixed effects of diet (C1, C2) and meal (M1, M2, M3, M4), and their related interaction, on the food and energy intake of dogs. The variable ‘dog’ was defined as a random term. In a similar manner, a linear mixed model was used to assess the fixed effect of diet (F1, F2) on the food and energy intake of cats, with the variable ‘cat’ being included as a random term. Given the design of the palatability studies, the fixed effects of diet (C1, C2 for dogs; F1, F2 for cats) and either meal (M1, M2) for dogs or day (D1, D2) for cats with their related interaction were assessed on food intake. The variables ‘dog’ and ‘cat’ were included as random terms in the model.

In each case, when residuals of a model were not normally distributed at an alpha risk level of 1% (Shapiro-Wilk and Kolmogorov-Smirnov tests), that output variable was rank-transformed prior to analysis to be treated in a non-parametric manner. Post-hoc analysis *P*-values were adjusted using Scheffe method to deal with alpha risk inflation linked to multiple comparisons. Unless indicated otherwise, all data are expressed as median (range). The level of significance was set at 5% for 2-sided analyses.

## Results

### Canine VFI studies

#### Study 1

Before the study, BW was 5.82 kg (3.96–10.46 kg), and was 6.09 kg (4.00–11.44 kg), after the study. Despite the small but significant increase in bodyweight (+0.12 kg [+2.1%, of starting BW], range −0.10 to +0.98 kg [−2.4% to +10.3%], *P* = 0.016), all dogs remained in ideal body condition (e.g. 5/9) throughout the study.

When food intake was assessed on an energy basis (Fig. 2a), a significant diet effect was evident (*P* = 0.032), with dogs consuming less of diet C1 (198 kcal/kg^0.75^ [144–268 kcal/kg^0.75^]) than of (C2: 206 kcal/kg^0.75^ [121–338 kcal/kg^0.75^]). Post-hoc analysis revealed the main difference in food intake to be at meal 2, where 42% less of C1 was eaten than C2 (*P* = 0.006). An interaction was also seen between the diet and meal effects (*P* < 0.001), with the evolution of food intake over the successive meals differing between the two diets. Specifically, a significant reduction of energy intake was observed between the second and third meals for both diets (*P* < 0.001), but between the first and second meals for diet C1 only (C1: *P* < 0.001; C2: *P* = 0.256). Nevertheless, an overall decrease in food intake between meal 1 and meal 4 was also evident for both diets (−86.5%, *p* < 0.001; −88.1%, *p* < 0.001 for diets C1 and C2, respectively).

When food intake was instead assessed on a gram weight basis (Fig. 2b), the significant dog (*P* = 0.016) and meal (*P* < 0.001) effects remained, but there was no longer a diet effect (total food intake on C1: 256 g grams [150–542 g]; total food intake on C2: 252 g [113–476 g]; *P* = 0.964). However, the diet-meal interaction was still evident (*P* < 0.001) with a significant gram weight reduction in food intake observed between the second and third meals for both diets (*P* < 0.001), but between the first and second meals for diet C1 only (C1: *P* < 0.001; C2: *P* = 0.960).

#### Study 2

Before the study, BW was 11.54 kg (9.46–14.16 kg), 11.48 kg (9.60–14.28 kg) after study period 1, and 11.34 kg (9.38–14.52 kg), after study period 2. Bodyweight did not change significantly in this time (*P* = 0.863), and all dogs remained in ideal body condition (e.g. 5/9) throughout.

**Fig. 3:**
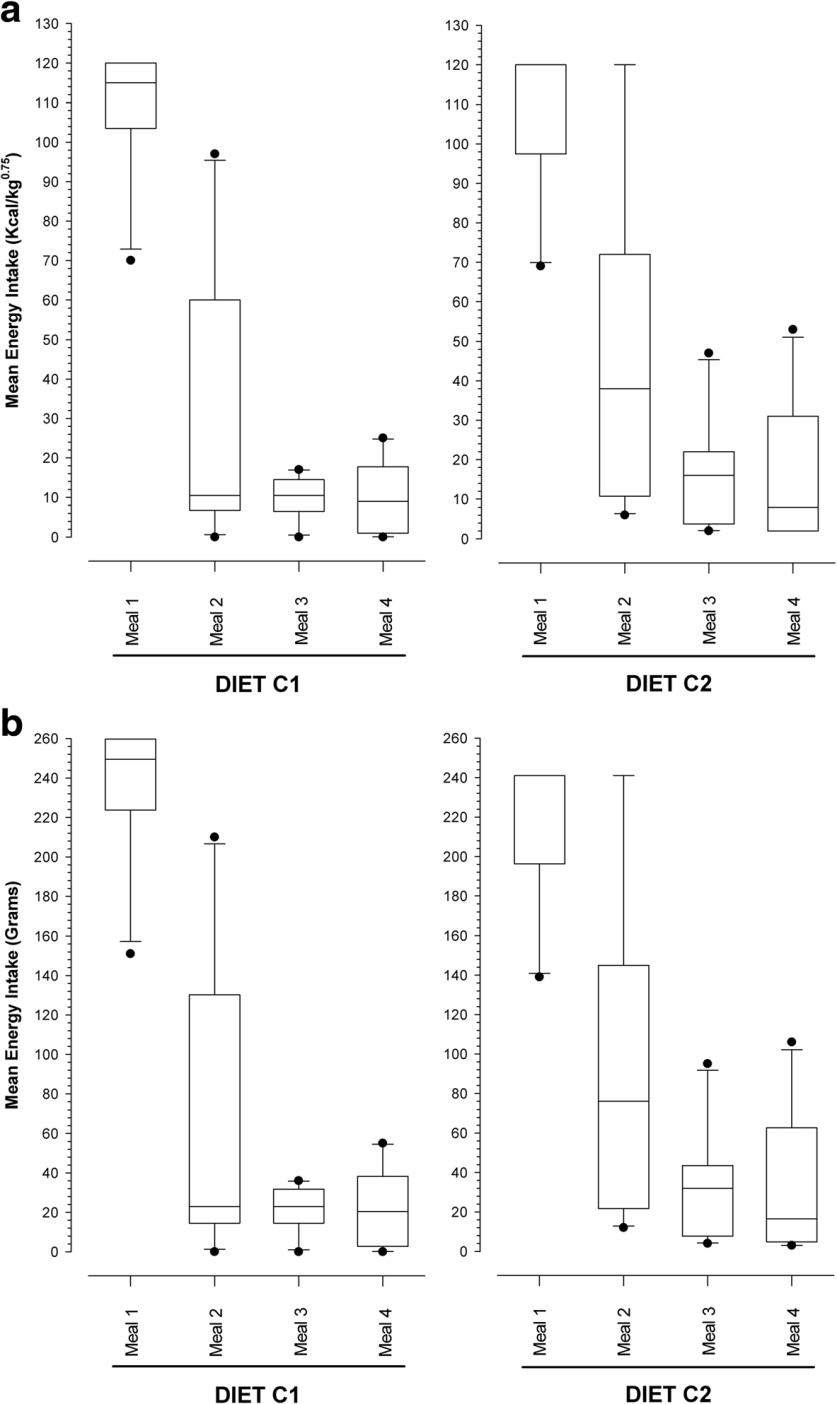
Box and whisker plots of sequential energy (**a**) and gram weight (**b**) intake dogs in the second canine voluntary food intake study (Study 2) where dogs were fed the two study diets (C1 and C2), over four meals. The boxes depict median (horizontal line) and inter-quartile range (top and bottom of box), the whiskers show the 10–90% range, and outliers are shown as separate points. **a** A significant reduction of energy intake was observed between the first and second (*P* < 0.001) and the second and third (*P* < 0.001) meals for both diets, but there was no difference in intake between the 3^rd^ and 4^th^ meals (*P* = 1.000). A diet effect was also evident (*P* = 0.019), with the main difference being a lesser intake at meal two for C1 compared with C2 (*P* = 0.006). **b** A significant reduction in gram weight intake of food was observed between the first and second meals for both diets (C1: *P* < 0.001; C2: *P* = 0.009), but not between either the other meals. Further, no difference in the gram weight intake of food was observed between diets (*P* = 0.255)

When food intake was assessed on an energy basis (Fig. 3a), a significant diet effect was again evident (*P* = 0.019) with dogs consuming less of diet C1 (147 kcal/kg^0.75^ [93–225 kcal/kg^0.75^]) than of diet C2 (189 kcal/kg^0.75^ [86–290 kcal/kg^0.75^]; *P* = 0.019). As with study 1, a significant meal effect was also observed (*P* < 0.001), with a significant reduction in intake occurring after each consecutive meal, except between the 3^rd^ and 4^th^ meals. Finally, a significant dog effect was also found (*P* = 0.046), but there was no diet-meal interaction (*P* = 0.434).

When food intake was instead assessed on a gram weight basis (Fig. 3b), the significant meal effect remained (*P* < 0.001), but neither the dog (*P* = 0.052) nor diet (total food intake on C1: 318 g [202–487 g]; total food intake on C2: 380 g [173–582 g]; *P* = 0.255) effects were evident. In contrast to the results expressed on an energy basis, a diet-meal interaction was evident (*P* = 0.023; diet C1: meal 1 vs. meal 2 *P* < 0.001; meal 2 vs. meal 3, *P* = 0.278; meal 3 vs. meal 4, *P* = 1.000; diet C2: meal 1 vs. meal 2 *P* = 0.009; meal 2 vs. meal 3, *P* = 0.069; meal 3 vs. meal 4, *P* = 1.000).

### Feline VFI study

Prior to analysis, data from one cat were excluded on account of malfunction of the electronic food scales. Body weight prior to and after the studies was 4.32 kg (2.66–5.88 kg) and 4.26 kg (2.67–5.81 kg), respectively. There was no change in BW (*P* = 0.067) over the study period, and there was no change in BCS for any cat during this time.

During the course of the study, a diet effect was found when data were expressed on an energy basis (*P* < 0.001), with intake on diet F1 (55 Kcal/kg^0.711^, 0–143 Kcal/kg^0.711^) being 17% less than intake when consuming diet F2 (66 Kcal/kg^0.711^, 41–158 Kcal/kg^0.711^). A significant cat effect was also evident (*P* = 0.023). When data were expressed on a gram weight basis, the cat effect remained (*P* = 0.023), but there was no longer a diet effect (F1: 51 g [0–127 g]; F2: 55 g [33–122 g]; *P* = 0.207).

### Palatability studies

In the canine palatability test, the median intake of diets C1 and C2 was 41 g (range 0–350 g) and 36 g (range 0–350 g), respectively. Total food intake (combined intake of C1 and C2 for each dog) during the study was 136 g (26–427 g). There was no significant meal effect (*P* = 0.914) and no significant difference in food consumption between diets was observed (*P* = 0.490). In the feline palatability test, the median intakes of diets F1 and F2 were 30 g (0–66 g) and 7 g (0-66 g), respectively. Total food intake (combined intake of F1 and F2 for each cat) was 40 g (18–133 g). No significant day effect was observed (*P* = 0.476), but there was a highly significant difference in consumption of the two diets (*P* < 0.001).

## Discussion

In the current study, performance (in terms of VFI and palatability) of different commercially available purpose-formulated canine and feline weight loss diets was assessed in groups of healthy dogs and cats in ideal body condition. There were significant differences in overall energy intake between the diets tested in both the canine and feline studies. These findings are important given that maximising satiety is a critical factor for any diet used in a controlled weight loss programme [16, 17].

The canine diets differed in energy content, macronutrient content, the sources of fibre, individual ingredients, and also in organoleptic properties. As a result, there could be various explanations for the observed differences. First, and most likely, the differences in energy intake could be due to differences in energy content because diet C1 was 8% less energy dense than diet C2. This explanation is supported by the fact that, when VFI was expressed on a gram weight basis (rather than on an energy basis), the diet effect was no longer evident. Against this, however, a diet-meal interaction was also observed: whilst, intake for both diets tended to decrease steadily across the four meals, differences in the pattern between diets was observed, most notably with a lower intake on diet C1 at meal 2. It is difficult to reconcile such a meal effect if the energy intake difference was simply due to relative energy dilution. Further, in a previous study with a similar design, the diet that was consumed least did not have the lowest energy content [22]. This suggests that factors in addition to energy dilution might be responsible for the observed differences in energy intake on the two diets. Other possible reasons could include differences in macronutrient content, specifically protein and fibre content, as previously demonstrated [17, 22]. Relative to energy content, diet C1 had 19% more protein and 21% more fibre than diet C2, which is equivalent to the differences between the 3 diets used in a previous study [22]. This again suggests that foods containing more protein and fibre have the best satiety, an observation supported by human studies [26–30].

As for the canine studies, no differences in VFI were seen between feline diets when measured by the gram weight, but cats consumed 17% less, of diet F1 compared with diet F2, when intake was expressed on an energy basis. Like the canine diets, the feline diets differed in energy (F1 15% less than F2) and total dietary fibre content (F1 35% more than F2). However, in contrast to the canine diets, protein content was similar between the feline diets, and diet F1 also contained 32% less dietary fat than F2. Finally, there were also differences in the type of fibre included and the ingredient lists for the two diets. Whatever the reason for the diet effect on voluntary energy intake, the results do suggest differences in the satiety effect between weight loss diets in cats, supporting the findings of other studies whereby the same diet resulted in less marked begging behaviour than other diets in obese cats during weight loss [16].

With regard to fibre type, the main fibre sources in the canine and feline diets where energy intake was least were vegetable fibres, beet pulp, psyllium and chicory pulp (F1 only), whilst the fibre used in the diets where energy intake was greatest was pea bran meal, tomato pomace, beet pulp, and powdered cellulose. Fibre types can differ greatly in their properties, leading to highly variable influences on water binding, gastric emptying, and the viscosity of the digesta, thus exerting different effects on VFI. Indeed, studies undertaken in humans have shown that psyllium improves satiety [31–33]. For instance, the vegetable fibre used in diet F1 contains cellulose with a high water binding capacity, and this could help delay gastric emptying explaining the improved satiety. More details about the exact fibre blends used for each diet might have shed light on their specific properties. However, since the diets used are sold commercially, such details constitute proprietary information and therefore are not publicly available. Therefore, it was not possible to fully assess the relative effects of fibre type and other factors (such as macronutrient content and energy density), and this is acknowledged as a study limitation. Nonetheless, the advantage of using commercially-available diets was the fact that the results would be more directly relevant to clinical practice.

One possible explanation for a difference in VFI between two diets, is if they differ in palatability and, for this reason, food preference tests were also performed. The palatability of the two canine diets was equivalent, whilst the feline diet that was least consumed was found to be significantly more palatable. In light of these findings, palatability differences amongst diets are not likely to account for study results, and the effect of the F1 diet on VFI in cats may well be even more pronounced given this superior palatability. In contrast, no differences in palatability were seen between the two canine diets, again suggesting that this is unlikely to be the reason for the differences in VFI between diets C1 and C2. However, it should be noted that this palatability study was conducted in Winter, whilst, all other studies (including the feline palatability study) were conducted in spring-summer. It is unclear whether this difference might have affected the results obtained.

Different designs were used to assess VFI in the canine and feline experiments. Dogs can consume large amounts of food in a single sitting, whilst cats prefer to consume food in multiple meals throughout the day, with each meal being small [34]. For this reason, the canine experiments involved assessing short-term VFI by monitoring food consumption kinetics in a 4-h period, based upon a design used in a previous study [22]. In contrast, daily VFI was measured in cats using automated food stations, again, as previously reported [23]. The use of such food stations, which recognised individual cats, allowed individual cats to consume food in whatever meal pattern they preferred during the study period, whilst ensuring that the amount consumed was accurately and precisely measured. In the authors’ opinion, the use of such devices is essential for assessing VFI in this species, and would recommend them for all future studies.

As with any study, a number of limitations must be considered in addition to those detailed above. First, studies used small groups of dogs and cats housed in colonies rather than pet dogs and cats in their home environment. Thus, results might not be generalisable to the larger pet population that would have greater inherent variability in terms of animal factors, environment and the fact that they would be client-owned. That said, the advantage of using colony animals was the fact that experimental conditions could be better controlled and study parameters such as food intake and palatability more precisely measured. Second, the replicate experiments for the canine VFI study were undertaken at different sites, using different dogs and housing conditions. Although the results were broadly similar, there was some variability observed. Third, also for the canine VFI studies, no adaptation period was included between the test periods for each. This might have affected the feeding kinetics of the study, although it is unclear as to whether any systematic bias resulted because the order in which diets were fed was arbitrarily decided.

A fourth study limitation was the fact that all of the VFI studies were short term in nature, and it is not known whether the satiating effect wanes when a restricted diet is fed continually. Similarly, the palatability studies were only conducted over two consecutive meal periods (two meals in a single day for dogs; two 22-h periods on consecutive days for cats), and thus did not assess whether taste preferences might have changed with time.

Finally, the study did not assess diet performance in overweight pet dogs and cats during energy restriction in order to induce controlled weight loss; instead, healthy research colony animals in optimal body condition were used and none of them lost weight during the study. Therefore, the results of the current study may not be generalisable to the target population. The main reason for our choice of research colony animals over pet animals was a far greater ability to control experimental conditions, thus improving accuracy of results and reducing the number of animals required to participate. Whilst not impossible, it would have been logistically difficult to perform similar studies in overweight pet dogs in their own homes. In this respect, the study population would inevitably have been far more variable, for example differing in the degree of obesity, energy restriction required for weight loss, and in terms of concurrent illness present [19]. There would also have been more variability in housing conditions with differences in ambient temperature, lighting, and space available. Husbandry practices would have differed markedly for example the timing and method of feeding, provision of water, the exercise undertaken, and also participation in play activity. Owner factors would also be a consideration, with concerns over compliance with the study protocol [14, 15, 18]. Moreover, there would likely have variability in experimental conduct when extrapolated to the home environment and a greater likelihood of errors made in the timing of meals and measurement of food consumption. Finally, the use pet animals would have introduced ethical considerations; although none of the procedures were invasive adverse effects making adverse effects on welfare unlikely, it is questionable as to whether the animals would have benefitted from participating in the study. All-in-all, therefore, despite the inevitable limitations of using healthy colony animals, this approach was preferred. Whilst caution should be exercised when generalising our results to the wider pet population, the results are nevertheless interesting, suggesting that diets C1 and F1 would perform better and reduce unwanted begging activity in pets animals, as seen in a previous field study [16]. Nonetheless, further studies would now be needed in order to assess these diets under field conditions in obese dogs and cats undergoing controlled weight loss.

## Conclusion

In summary, the results of the experiments in the current study have demonstrated differences in voluntary energy intake in both cats and dogs when consuming commercially available weight loss diets. Possible explanations for the superior performance of diet C1 (vs. diet C2) include decreased energy content, increased protein and fibre content, and/or using psyllium and beet pulp as the fibre sources. In contrast, the possible explanations for the superior effect of diet F1 (vs. diet F2) include decreased energy and fat content, increased dietary fibre content, and/or using psyllium and chicory pulp as the main fibre sources. Further studies are now recommended so as to assess the performance of these weight loss diets in obese pet dogs and cats during a controlled weight loss programme.

## Additional file

Additional file 1: Computer spreadsheet containing data from all studies. (XLSX 36 kb)

## Abbreviations

BCS: Body condition score
BW: Body weight
MBW: Metabolic body weight
MER: Metabolisable energy required for maintenance
NFE: Nitrogen-free extract
ONIRIS: Nutrition and Endocrinology Unit, National Veterinary School of Nantes
SAS: Statistical analysis systems
VFI: Voluntary food intake
