# Erratum to: Comparison of voluntary food intake and palatability of commercial weight loss diets in healthy dogs and cats

**DOI:** 10.1186/s12917-017-0959-x

**Published:** 2017-01-26

**Authors:** Marie Anne Hours, Emmanuelle Sagols, Ariane Junien-Castagna, Alexandre Feugier, Delphine Moniot, Ingrid Daniel, Vincent Biourge, Samuel Serisier, Yann Queau, Alexander J. German

**Affiliations:** 1 Royal Canin Research Center, Aimargues, France; 2 Unité de Nutrition et d’Endocrinologie, Oniris, Nantes, France; 3 Institute of Ageing and Chronic Disease, University of Liverpool, Leahurst Campus, Chester High Road, Neston, Wirral CH64 7TE UK; 4 Université de Nantes, Faculty of Dental Surgery, 1 Place Alexis Ricordeau, 44042 Nantes, France; 5 Institute of Veterinary Science, University of Liverpool, Leahurst Campus, Chester High Road, Neston, CH64 7TE UK

## Erratum

The original article [1] contains an error whereby the author Samuel Serisier’s name is displayed incorrectly as ‘Serisier Samuel’.

This should instead read ‘Samuel Serisier’.
